# A Current Review of Cypermethrin-Induced Neurotoxicity and Nigrostriatal Dopaminergic Neurodegeneration

**DOI:** 10.2174/157015912799362779

**Published:** 2012-03

**Authors:** Anand Kumar Singh, Manindra Nath Tiwari, Om Prakash, Mahendra Pratap Singh

**Affiliations:** 1 Indian Institute of Toxicology Research (Council of Scientific and Industrial Research), M. G. Marg, Post Box - 80, Lucknow - 226 001, India Varanasi - 221 005, India; 2 Banaras Hindu University, Varanasi - 221 005, India

**Keywords:** Cypermethrin, model systems, neurotoxicity, neurodegeneration, Parkinson’s disease, pesticides.

## Abstract

Cypermethrin, a class II pyrethroid pesticide, is used to control insects in the household and agricultural fields. Despite beneficial roles, its uncontrolled and repetitive applications lead to unintended effects in non-target organisms. Cypermethrin crosses the blood-brain barrier and induces neurotoxicity and motor deficits. Cypermethrin prolongs the opening of sodium channel, a major site of its action, leading to hyper-excitation of the central nervous system. In addition to sodium channel, cypermethrin modulates chloride, voltage-gated calcium and potassium channels, alters the activity of glutamate and acetylcholine receptors and adenosine triphosphatases and induces DNA damage and oxidative stress in the neuronal cells. Cypermethrin also modulates the level of neurotransmitters, including gamma-aminobutyric acid and dopamine. It is one of the most commonly used pesticides in neurotoxicology research not only because of its variable responses depending upon the doses, time and routes of exposure and strain, age, gender and species of animals used across multiple studies but also owing to its ability to induce the nigrostriatal dopaminergic neurodegeneration. This article describes the effect of acute, chronic, developmental and adulthood exposures to cypermethrin in experimental animals. The article sheds light on cypermethrin-induced changes in the central nervous system, including its contribution in the onset of specific features, which are associated with the nigrostriatal dopaminergic neurodegeneration. Resemblances and dissimilarities of cypermethrin-induced nigrostriatal dopaminergic neurodegeneration with sporadic and chemicals-induced disease models along with its advantages and pitfalls are also discussed.

## INTRODUCTION

Cypermethrin (chemical abstract (CA) name: (***RS***)-α-cyano-3-phenoxybenzyl(1***RS***,3***RS***;1***RS***,3***SR***)-3-(2,2-dichlorovinyl)-2,2-dimethylcyclopropanecarboxylate and international union of pure and applied chemistry (IUPAC) name (***RS***)-alpha-cyano-3-phenoxybenzyl (1RS)*cis-*trans-3-(2,2-dichlorovinyl)-2,2-dimethyl-cyclopropane carboxylate) is one of the most widely used synthetic insecticides for agricultural and domestic purposes, globally [1]. In general, insecticide sprayers and industrial worker are rarely exposed to single insecticides in their day-to-day life. Mostly, they are exposed to multiple classes of pesticides altogether. Several epidemiological and experimental studies have been performed to assess the health risks associated with cypermethrin exposure and measured cypermethrin level in the blood and urine of the pesticides sprayers and exposed individuals [2-8]. Cypermethrin has been identified as one of the important constituent pesticides associated with human health risks [8]. 

Cypermethrin is a class II synthetic pyrethroid pesticide and crosses the blood brain barrier and exerts neurotoxicity in the central nervous system and also induces motor deficits. Pyrethroids, including, cypermethrin extend the opening of sodium channels in the central nervous system leading to hypo-polarization and hyper-excitation of the neurons [9-11]. Short-term neurotoxicity caused by cypermethrin is primarily mediated through hyper-excitation of the central nervous system [9-11]. Additionally, cypermethrin induces neurotoxicity by modulating the level of gamma-aminobutyric acid (GABA) [12]. Furthermore, cypermethrin-mediated neurotoxicity is contributed by its ability to induce free radical generation [13, 14]. Since oxidative stress critically contributes to the nigrostriatal dopaminergic neurodegeneration, cypermethrin could be considered as one of the most relevant pesticides, which possibly implicates in Parkinson’s disease (PD) pathogenesis [15]. Despite strong debate over the issue, investigations have been performed to elucidate the deleterious effects of cypermethrin on the nigrostriatal tissues. Interestingly, concurrence on the issue could not be achieved from the results obtained across various investigations [16, 17]. A few reports have highlighted the adverse effects of cypermethrin leading to the nigrostriatal dopaminergic neurotoxicity [16, 17], while others have shown that it enhances the nigrostriatal dopaminergic neurodegeneration only when administered in combination with any other chemicals or stimuli, which trigger neurodegeneration [17]. Lack of consensus among these studies could be easily explained on the basis of different experimental paradigms employed therein. Firstly, the doses and routes of exposure used across the studies were not the same. Secondly, its neurodegenerative potential was assessed in experimental animals exposed to it only for three weeks or even shorter periods [16, 17]. Additionally, these investigations were performed after acute to sub-acute or very low to mild doses during developmental or adulthood stages [15-17]. The effects of cypermethrin on dopaminergic neurons in adult animals after multiple exposures from low to high doses and from short to long-term exposures have been recently reported [18, 19]. Short-term exposure to cypermethrin did not produce neurodegeneration, as observed for two other commonly used pesticides, maneb (CA name: [1,2-ethanediylbis(carbamodithioato)(2-)]manganese and IUPAC name: manganese ethylene-1,2-bisdithiocarbamate)- and paraquat (CA name: 1,1’-dimethyl-4,4’-bipyridinium dichloride and IUPAC name: 1,1’dimethyl-4,4’-bipyridinediium dichloride) [20, 21]. Prolonged exposure to moderate doses induced the nigrostriatal dopaminergic neurodegeneration in adult animals and the response was more pronounced in the animals pre-exposed to very low doses during the critical periods of development [18, 19]. Developing nervous system is expected to be highly sensitive and may act as a preferential target for pesticides because the development of dopaminergic neurons mainly occurs during postnatal periods [22]. Loss of invisible regulations of dopaminergic neurons during postnatal exposure to pesticides leads to irreversible changes in the nigrostriatal system, which appears in the adults, when re-challenged [18, 19]. Since the selective degeneration of the nigrostriatal dopaminergic neurons is one of the major hallmarks of PD [23], the contribution of cypermethrin in PD pathogenesis is inevitable and worthwhile to discuss in this article. The article presents a brief synopsis of cypermethrin-mediated acute and chronic neurotoxicities in experimental animals, especially rodents. This review offers a glimpse of cypermethrin-mediated changes, which raise the need to assess its impact in the pathogenesis of sporadic PD. Finally, possible implications of cypermethrin-induced nigrostriatal dopaminergic neurodegeneration in sporadic PD, its advantages and limitations over other model systems used in PD research along with its future perspectives have been discussed in this review.

## CYPERMETHRIN-INDUCED NEUROTOXICITY 

Cypermethrin causes toxicity in many parts of brain, depending upon the doses, time and routes of exposures. Cypermethrin-mediated toxicity appears in experimental animals at all study levels, beginning from the biochemical to anatomical and molecular to phenotypic. 

## NEUROMUSCULAR AND NEUROBEHAVIORAL FUNCTIONS

Effects of cypermethrin on neurobehavioral indices have been a matter of intense interest to neurotoxicologists. Although cypermethrin did not produce visible changes in the neurobehavioral indices after acute oral exposure (150 mg/kg); at 1/10^th^ of the LD50, it induced skeletal muscle contraction in the hind limbs without any signs of dyskinesia, tremor and movement incoordination [24-27]. In general, acute intoxications to higher doses did not enhance skeletal muscle contraction, however, an increase in extensor tone, rolling gait and movement incoordination were observed, which could lead to tremors [24, 26, 27]. Oral administration of cypermethrin led to a sequence of visible motor symptoms along with chewing, licking and salivation. Furthermore, cypermethrin caused stayed limbs, increased foot splay, reduced arousal and reduced response to touch pinches [25]. Cypermethrin reduced limb grip strengths while increased the tremor intensity in a dose dependent manner [25], the two major characteristics of chemicals-induced PD. Contrary to the inconsistent pattern of motor functions at various doses, cypermethrin produced consistent increase in latency and decrease in sensitization and amplitude [28]. Cypermethrin also increased the reinforcement rate required to maintain half-maximal response, however, at higher doses, it influenced the relationship between reinforcement rate and performance [29].

Cypermethrin also exhibits developmental neurotoxicity in experimental animals (Table **1**). Newborns of cypermethrin-exposed mice (exposed before mating) showed reduced body weights and the reductions were found more pronounced in the newborns of mice exposed to higher doses of cypermethrin [30]. Cypermethrin is reported to induce the motor incoordination and clinical signs in the first generation progeny. However, delay in the development of ear pinna detachment, down appearance and eye opening are reported in the pups of high doses cypermethrin-treated mothers [30]. 

## GABAERGIC NEURONS

Several reports have highlighted that cypermethrin antagonizes GABA, a few investigations contradicted the same while some other studies have found decreased GABA level in brain of the animals exposed to high concentration of cypermethrin/class II pyrethroids [12]. Decreased GABA level in the cypermethrin treated rats was observed at doses more than 145mg/kg owing to its decreased synthesis or increased catabolism [12]. Since short-term exposure to cypermethrin did not alter GABA [12], the effect of long-term moderate exposure to cypermethrin on GABA was not measured in the nigrostriatal tissues [18]. 

## SEROTONERGIC NEURONS

Pyrethroids in general reduce serotonin and its metabolites after sub acute exposure in various parts of brain but more pronounced depletions are reported in the frontal cortex [31]. Although higher doses of pyrethroids enhanced the intensity of serotonin and its metabolites in the rat brain [31], its chronic treatments at moderate doses did not produce significant changes in serotonin level in the nigrostriatal tissues [18]. 

## ACETYL-AND BUTYRYL- CHOLINESTERASE ENZYMES 

Oral exposures to cypermethrin at low doses did not alter the acetyl- and butyryl- cholinesterase activities, while higher doses markedly reduced the levels in a dose dependent manner [32]. The reduced acetyl cholinesterase activity also produced harmful effects on the muscular and nervous systems [32].

## DOPAMINERGIC NEURONS

**Fig. (1):**
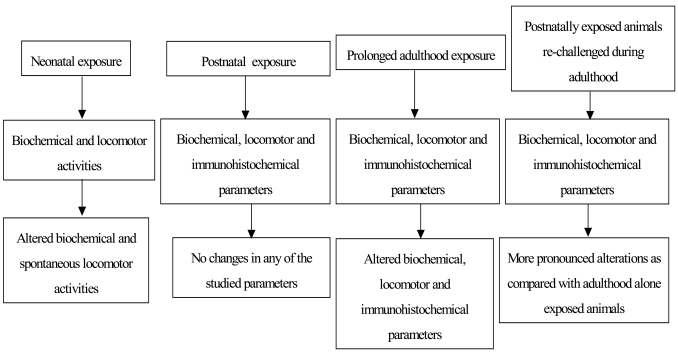
The salient findings of a few main studies [15, 18, 19, 66], which have shown the effects of cypermethrin on the nigrostriatal dopaminergic neurons.

Cypermethrin readily enters the brain and induces oxidative stress leading to dopaminergic neurotoxicity [15]. Due to its ability to produce deleterious effects in the nigrostriatal dopaminergic system, cypermethrin contributes to PD pathogenesis [15, 18]. An altered level of dopamine and its metabolites is reported in animals, which were exposed to cypermethrin for short-term study (Fig. **1**). Although animals exposed to it for shorter time did not show any symptoms of PD, co-exposures with MPTP (CA name: 1-methyl-4-phenyl-1,2,3,6-tetrahydropyridine and IUPAC name: 1-methyl-4-phenyl-3,6-dihydro-2H-pyridine) or 6-hydroxydopamine (6-OHDA) were found to enhance PD like features [17]. Low doses of cypermethrin (5 and 10 mg/kg body weight) did not alter the major indices of the nigrostriatal dopaminergic neurodegeneration in adult animals [18]. Recently, alteration in the levels of dopamine and its metabolites and loss of tyrosine hydroxylase positive cells in the nigrostriatal tissues and impaired motor behavior of treated animals were observed after prolonged exposure to cypermethrin at moderate doses [18, 19] (Fig. **1**). Animals exposed to cypermethrin during developmental periods at a very low dose followed by moderate dose adulthood re-exposure exhibited more pronounced changes in the neurodegenerative indices than that of adulthood alone treated animals [18, 19] (Table **2**). The enhanced response could be due to irreversible and invisible effects in the neonatal brain that appear in the adults when re-challenged, as the neonatal brain undergoes numerous biological changes during critical periods of development and acquires many new motor and sensory abilities, which transform it to the mature adult brain [33]. Post-natal periods are also critical for the synthesis of brain lipids and protein turnover that are at the highest levels during this time [34, 35].

## MECHANISMS OF CYPERMETHRIN-MEDIATED NEUROTOXICITY 

### Oxidative Stress

The oxidative stress is implicated in the cypermethrin-mediated neurotoxicity. The major contributors of oxidative stress are excessive production of reactive oxygen species (ROS) and reactive nitrogen species in the cells or tissues exposed to cypermethrin or reduced level of components of the antioxidant machinery. Oral or intraperitoneal administration of cypermethrin produces oxidative stress in the neuronal system [14, 19]. Despite significant changes in the expression of a few isoforms of glutathione-S-transferase (GST), no significant alteration in total GST activity was observed after moderate doses and long-term exposures [19]. Cytochrome P450 2E1 (CYP2E1) is recognized as one of the major contributors involved in cypermethrin metabolism leading to generation of ROS and oxidative stress *via *mixed function oxidase [14, 19, 36].

## DNA DAMAGE 

Cypermethrin causes DNA damage and reduces mitotic and nuclear divisions. Micronuclei assay showed that cypermethrin at lower concentrations induces DNA damage in human lymphocytes [37]. At lower concentrations, it increases DNA damage in the basal ganglion in a dose-dependent manner. Cypermethrin crosses placental barrier and produces harmful effects in embryo even at very low concentrations. Exposure to cypermethrin reduces DNA content and may lead to mutation, especially germline mutation leading to teratological deformities [32, 38, 39]. Cypermethrin produces mutagenicity and genotoxicity by interacting with DNA metabolic processes, sister chromatid exchange and free radical generation machinery [40, 41]. Contrary to it, cypermethrin was found to affect cell cycle leading to reduced proliferative rate index without any changes in chromosomal aberration or sister chromatid exchange in human peripheral lymphocytes [42]. Its dermal exposure caused repetitive firing of sensory nerve endings leading to systemic signs characterized by dizziness, headache and disturbances in consciousness, muscular fasciculation, convulsive attacks and coma [43, 44]. 

## ION CHANNELS

The neurotoxic responses of cypermethrin are mainly mediated by the modulation of ion channels. Cypermethrin modulates various ion channels, including sodium and chloride channels. Since one of the primary targets for cypermethrin is insect voltage-gated sodium channel (VGSC), it is expected that mammalian sodium channels and receptors regulated by it can also act as primary targets for toxicity in humans. Other main channels and receptors, which are influenced by cypermethrin include, chloride channels, voltage-gated calcium channels (VGCC), potassium channels, GABA receptors, glutamate receptors, acetylcholine receptors and ATPases.

## VOLTAGE-GATED SODIUM CHANNEL (VGSC)

The major mode of action of cypermethrin like other class II pyrethroids is the disruption of VGSC function [45]. Disruption of sodium channel function is mediated by specific binding sites. Initially, cypermethrin binds at Phe1519 residue, which could induce conformational changes necessary for the formation of an optimal binding site [45]. Binding of cypermethrin initially slows the activation or opening of the channel followed by slowing the rate of channel inactivation and shifting of membrane potential towards more hyperpolarized stage, which is required for the channel to activate [46]. Consequently, sodium channels open at more hyperpolarized potentials and remain open for longer, allowing more sodium ions to cross and depolarize the neuronal membrane leading to hyper excitability to the point at which generation of the action potentials is not possible. 

Ion channels are essential components of the living cells, which are known to perform normal biological functions [47]. Cypermethrin modulates the function of VGSC [45]. Cypermethrin binds with and disrupts the correct functioning of ion channel leading to death of insects. Cypermethrin alters the membrane potential and sodium ion permeability in time of exposure dependent manner. Fortunately, sodium channels found in mammals are less sensitive to cypermethrin as compared with insects [48]; therefore, threat to human population is not as much as the concern arose. Pyrethroids, especially cypermethrin, delay the inactivation of VGSC and prolong sodium channel opening and cause repetitive firing of action potentials leading to hypo-polarization and hyper-excitation of the nervous system [9-11]. 

## VOLTAGE-GATED CALCIUM CHANNEL (VGCC)

Unlike sodium channel, cypermethrin alters the kinetics and calcium influx by the inhibition of VGCC [49], which regulates the protein kinases and phosphatases, the key enzymes involved in the signal transduction pathways. Inositol triphosphate (IP3)-mediated calcium influx is regulated by phosphorylation reaction arbitrated by Ca^2+ ^dependent serine/threonine phosphatase (calcineurin) through IP3 receptor- peptidyl-prolyl cis-trans isomerase FK-506 binding protein BP 12 complex. Since cypermethrin is one of the strong inhibitors of calcineurin, therefore, alters the channel dependent Ca^2+ ^influx leading to reduced cellular calcium level and impaired release of neurotransmitter [50, 51]. 

Owing to resemblances between sodium and calcium channels, the effect of cypermethrin on VGCC is not an unexpected phenomenon and therefore has been assessed in many studies. VGCC play critical role in nerve cell excitability, calcium homeostasis, synaptic signaling and modulation of gene expression [52-55]. At lower concentrations, cypermethrin did not produce any alterations; however, it induced delay in phosphorylation of calcineurin and inhibition of calcium channel at higher concentrations [50]. 

## POTASSIUM CHANNELS

Cypermethrin alters the activity of delayed-rectifier voltage-dependent potassium channel and potassium ion transport across synaptosomes, which regulates the neuronal excitability and ultimately leads to neurotoxicity [56, 57]. 

Potassium current is one of the main targets of cypermethrin, which causes neurotoxic effects in many neurons [58]. Cypermethrin mediates neurotoxicity owing to its potential to modify the performance of potassium channel leading to an alteration in the activation potential. Delayed-rectifier voltage-dependent potassium channel regulates diverse aspects of neuronal excitability. Cypermethrin delays the function of this channel at lower concentrations; however, at higher concentrations, it inactivates potassium current [58]. 

## CHLORIDE CHANNELS

GABA is one of the most common targets of class II pyrethroids, including cypermethrin and regulates the chloride channels. Cypermethrin suppresses the open state of voltage-gated chloride channels and inhibits GABA dependent uptake of chloride ions [59-60], leading to hyper-excitability and neurotoxicity symptoms [61]. 

GABA neurotransmitter is one of the most predominating neurotransmitters, which regulates chloride channels in brain. Cypermethrin effectively suppressed the open state of voltage-gated chloride channel and inhibits GABA dependent chloride uptake at higher concentrations; however, no inhibition was observed below 25mg/kg doses [60-62]. Cypermethrin mediated inhibition of chloride channel is known to produce minor tremors, depression, grinding of teeth, hyperesthesia, spastic paralysis and sunken eyes, etc., in a dose dependent manner [61]. Cypermethrin-mediated neurotoxicity seems to arise from excitability disturbance is further evidenced from its ability to inhibit the activity of acetyl cholinesterase maximally in the brain as compared with other organs leading to decreased cholinergic transmission and consequent accumulation of neurotransmitter acetylcholine resulting in the termination of nerve impulses [63]. 

## SIMILARITIES AND DISSIMILARITIES WITH OTHER KNOWN RODENT MODELS USED TO STUDY THE NIGROSTRIATAL DOPAMINERGIC NEURODEGENERATION: ADVANTAGES AND LIMITATIONS 

Neurodegeneration is mediated by the inhibition of antioxidant defense system, incurring oxidative damage to cytosolic proteins and by the inhibition of mitochondrial electron transport chain [23]. Most of the neurotoxicants, including cypermethrin, induce oxidative stress in brain by inhibiting antioxidants or generating free radicals. Cypermethrin, like rotenone (CA name: [2R-(2alpha,6aalpha,12aalpha)]-1,2,12,12a-tetrahydro-8,9-dimethoxy-2-(1-methylethenyl)[1]benzopyrano[3,4-b]furo[2,3-h]benzopyran-6(6aH)-one and IUPAC name: (2*R*,6a*S*,12a*S*)-1,2,6,6a,12,12a-hexahydro-2-isopropenyl-8,9-dimethoxychromeno[3,4-*b*]furo[2,3-*h*]chromen-6-one) and many other model systems, is not very specific to dopaminergic neurons in brain; however, in the nigrostriatal system its preferential target is dopaminergic neurons [18]. Phenotypic features and preferential and selective degenerations of dopaminergic neurons of the nigrostriatal tissues show almost similar results [18], as observed with other model systems. Mitochondria based mechanism is proposed for MPTP, paraquat, maneb and rotenone, as these chemicals selectively inhibit either mitochondrial complex-I or III and promote the generation of reactive oxygen species [64, 65]. Mitochondrion is known as a point of convergence between the chemicals-induced neurotoxicity and sporadic PD [65]. Although cypermethrin induces dopaminergic neurodegeneration in adult rats after prolonged exposure [18], the contribution of mitochondrial proteins in its neurotoxicity is not yet properly understood. Most of the toxins induce dopaminergic neurodegeneration after short-term exposure but cypermethrin induces the same after prolonged exposure. This phenomenon makes this system ideal to study sporadic PD unlike many other model systems, except maneb- and paraquat-model up to some extent. If epidemiological investigations and molecular bases in this animal model validate the similarity with sporadic disease, it may lead to better understanding of disease pathogenesis and development of proper therapeutic strategies to encounter PD. 

## HOW CLOSE AND HOW FAR TO SPORADIC PD?

Despite being the second most common neurodegenerative disease, the exact mechanisms of pathogenesis and effective therapies to cure PD are not yet clearly known [65]. Unlike most of the model systems, cypermethrin-induced nigrostriatal neurodegeneration follows the slow and progressive neurodegeneration like maneb- and paraquat-induced mouse model of PD. The main mechanism implicated in the nigrostriatal dopaminergic neuronal death has been oxidative stress and cypermethrin also induces oxidative stress [13, 14, 18, 19]. Cypermethrin induces the nigrostriatal dopaminergic neurodegeneration and behavioral deficits, as the result of slow and progressive loss of dopaminergic neuronal cells, one of the specific hallmarks of sporadic PD [66]. Cypermethrin like rotenone, causes neurotoxicity in many parts of brain, however, its preferential target in the nigrostriatal region remains dopaminergic neurons [18]. This drawback is not only for cypermethrin, as non-selective responses of paraquat, MPTP and 6-OHDA due to mitochondrial injury result into lesions in many parts of brain, including hippocampus, in addition to the nigrostriatal dopaminergic neurons [67-70]. Cypermethrin induces neurodegeneration only after long-term exposure (12 weeks), if it mimics with sporadic etiology at molecular and epidemiological levels, the system could be more relevant to humans as compared with other model systems [18, 66]. 

## FUTURE DIRECTIONS

Cypermethrin is one of the most widely used insecticides, therefore, assessing the molecular mechanism of its neurotoxicity leading to neurodegeneration, using genome and proteome wide approaches along with genetic and molecular interventions, would be worthwhile. Genomic, proteomic and genetic approaches possibly offer clues to identify novel or unique genes and proteins specific to cypermethrin as well as specific to common neurodegeneration induced by various chemical entities. Validation of molecular fingerprints using other animals and strains may help in understanding the animal specificity to cypermethrin, if any. The roles of inflammatory mediators and other secondary signaling molecules, which finally lead to the molecular mechanism of the selective degeneration, need to be investigated. Role of mitochondria needs to be extensively investigated in this model system, as several mutated genes are known, which encode mitochondrial proteins and alter PD risk [65]. Since most of the chemicals inducing PD act through the inhibition of complex I-III of the electron transport chain, mitochondrial proteomics is expected to offer some novel findings. As humans are exposed to cypermethrin, which induces PD like features and the functions of many mitochondrial proteins are not yet known and several proteins implicated in PD are localized in or interact with mitochondria [65, 71, 72], therefore, mitochondrial alterations, membrane potential changes and reactive oxygen species generation along with the functions of mitochondrial proteins associated with cypermethrin-induced neurodegeneration need to be checked precisely. Mitochondrial proteomics will not only predict the involvement of classical pathways known for other pesticides but also identify novel proteins that follow alternate routes of neurodegeneration. Mitochondrial proteomics may offer the suitability and appropriateness of cypermethrin-induced nigrostriatal dopaminergic neurodegeneration over other model systems in order to understand the elusive aspects of sporadic PD and to assess the efficacy of novel neuroprotective agents.

## CONCLUSIONS

Cypermethrin-induced neurotoxicity is a matter of concern, as humans are exposed to it, in their day-to-day life. Despite variability in neurotoxicity responsiveness due to various contributory factors, such as doses, time and routes of exposure as well as model organisms, potential of cypermethrin to induce the nigrostriatal dopaminergic neurodegeneration has been a major concern in neuroscience research. Cypermethrin-induced changes in the central nervous system of experimental animals warrant multi-faceted studies, which can help in assessing the advantages and pitfalls that could be exploited further in knowledge generation on a few elusive aspects of sporadic PD and developing its counteractive measures. 

## Figures and Tables

**Table 1.** Cypermethrin-Mediated Developmental Neurotoxicity

Model System	Dose and Route of Exposures	Time of Exposure	Neurotoxic Effects	References

Wistar Rat	15 mg/kg, oral	Acute	Delayed surface righting, eye opening, fur development, incisor eruption and pinna detachment	[73]
			No effect on motor activity	
			Altered regional polyamine levels	

Wistar Rat	15 mg/kg, intraperitoneal	Postnatal days 5-19	No change in dopamine or its metabolites level, neurobehavioral indices and number of dopaminergic neurons two months after exposure	[18, 19]

Druck Rey Rat	5 mg/kg, oral	Postnatal days 13, 17 or 30	Increased the blood brain barrier permeability, which was recovered by day 60 after withdrawal on day 18	[74]

	2.5 mg/kg, oral	Postnatal days 10-17	Increased the blood brain barrier permeability	

Charles Wistar Rat	15 mg/kg, oral	Gestation days 5-21 Postnatal days 1-15	No change on dam weight, food and water intake, gestation length, offspring number and sex ratio	[75]
			No effect on monoamine oxidase, sodium potassium ATPase and acetylcholine esterase activities	
			No effect on spiroperidol binding	
			Decreased sodium potassium ATPase and acetylcholine esterase activities up to 3 weeks of age	
			Increased spiroperidol binding	

**Table 2.** Cypermethrin-Mediated Adult Neurotoxicity

Model Organism	Dose and Route of Exposure	Exposure Time	Neurotoxic Effects	References

Sprague-Dawley rats	15 mg/kg, oral	15 days	No change in the dopaminergic neuronal system	[17]
	75 mg/kg, oral		Induced appearance of abnormal behavior i.e., severe convulsive movement, salivation after a few hours of exposure	
	15 mg/kg, oral to 6-OHDA pre-exposed		Reduced the number of dopaminergic neurons in the substantia nigra and number of forepaw adjusting steps	

Wistar rats	145 mg/kg and 14.5 mg/kg, oral	One dose	Ataxia after a few hours of exposure	[12]
			Potentiated the pentobarbitone-induced sleeping time	
			Enhanced convulsion in pentylenetetrazole co-treated rats	

Wistar rats	15 mg/kg, intraperiton-eal	12 weeks, 24 doses	Decreased locomotor activity, dopamine and its metabolites levels and tyrosine hydroxylase-positive cells	[18]
			No change in serotonin level and glutamic acid decorbaxylase-positive cells in the nigrostriatal tissues	
